# Genotypic–Phenotypic Correlations of Hereditary Hyperferritinemia-Cataract Syndrome: Case Series of Three Brazilian Families

**DOI:** 10.3390/ijms241511876

**Published:** 2023-07-25

**Authors:** Olivia A. Zin, Luiza M. Neves, Daniela P. Cunha, Fabiana L. Motta, Bruna N. S. Agonigi, Dafne D. G. Horovitz, Daltro C. Almeida, Jocieli Malacarne, Ana Paula S. Rodrigues, Adriana B. Carvalho, Cinthia A. Rivello, Rita Espariz, Andrea A. Zin, Juliana M. F. Sallum, Zilton F. M. Vasconcelos

**Affiliations:** 1 Department of Ophthalmology, Universidade Federal de São Paulo, São Paulo 04039-032, Brazil; olivia.zin@unifesp.br (O.A.Z.); anapaulasilveriorodrigues@gmail.com (A.P.S.R.); juliana@pobox.com (J.M.F.S.); 2 Instituto Brasileiro de Oftalmologia, Rio de Janeiro 22250-040, Brazil; cinthiarivello@gmail.com (C.A.R.); andrea.zin@fiocruz.br (A.A.Z.); 3 Instituto Fernandes Figueira-Fundação Oswaldo Cruz, Rio de Janeiro 22250-020, Brazil; luiza.macneves@gmail.com (L.M.N.); danielapradocunha@gmail.com (D.P.C.); bagonigi@gmail.com (B.N.S.A.); dafne.horovitz@fiocruz.br (D.D.G.H.); daltro.castelar.jr@gmail.com (D.C.A.J.); jocielimlc@gmail.com (J.M.); 4 Department of Ophthalmology, Universidade do Estado do Rio de Janeiro, Rio de janeiro 20551-030, Brazil; 5 Instituto de Genética Ocular, São Paulo 04552-050, Brazil; fabiana.louise@gmail.com; 6 Instituto Nacional de Cardiologia, Rio de Janeiro 22240-006, Brazil; carvalhoab@biof.ufrj.br; 7 Instituto Catarata Infantil, Rio de Janeiro 22250-040, Brazil; 8 Department of Hematology, Hospital Federal Cardoso Fontes, Rio de Janeiro 22745-130, Brazil; respariz@gmail.com

**Keywords:** hyperferritinemia, cataracts, pediatric cataracts, hemochromatosis, genotype–phenotype correlation

## Abstract

Hereditary hyperferritinemia-cataract syndrome (HHCS) is a rare, frequently misdiagnosed, autosomal dominant disease caused by mutations in the *FTL* gene. It causes bilateral pediatric cataract and hyperferritinemia without iron overload. The objective of this case series, describing three Brazilian families, is to increase awareness of HHCS, as well as to discuss possible phenotypic interactions with concurrent mutations in *HFE*, the gene associated with autosomal recessive inheritance hereditary hemochromatosis. Whole-exome sequencing was performed in eight individuals with HHCS from three different families, as well as one unaffected member from each family for trio analysis—a total of eleven individuals. Ophthalmological and clinical genetic evaluations were conducted. The likely pathogenic variant c.-157G>A in *FTL* was found in all affected individuals. They presented slowly progressing bilateral cataract symptoms before the age of 14, with a phenotype of varied bilateral diffuse opacities. Hyperferritinemia was present in all affected members, varying from 971 ng/mL to 4899 ng/mL. There were two affected individuals with one concurrent pathogenic variant in *HFE* (c.187C>G, p.H63D), who were also the ones with the highest values of serum ferritin in our cohort. Few publications describe individuals with pathogenic mutations in both *FTL* and *HFE* genes, and further studies are needed to assess possible phenotypic interactions causing higher values of hyperferritinemia.

## 1. Introduction

Hereditary hyperferritinemia-cataract syndrome (HHCS) (OMIM 600886) is a rare autosomal dominant disease caused by mutations in the ferritin light chain gene (*FTL*). It was first described in 1995 [1,2], while detailed documentation of the cataract phenotype only occurred in 2001 [3]. About 120 families have been reported, according to Cat-Map, the online database for inherited and age-related forms of cataract in humans and mice [4].

A single study attempted to calculate its prevalence, finding a minimum prevalence of 1/200,000 in Australia, although most likely an underestimation [5]. Ophthalmologists do not routinely order serum ferritin in pediatric cataract investigations, and mutations have been described which lead to lower levels of hyperferritinemia, which could result in clinically insignificant cataracts.

Ferritin synthesis is regulated by iron availability via interactions between iron regulatory protein (IRP) and the iron-regulatory element (IRE) of the *FTL* gene. Mutations in the light chain subunits of ferritin (L-ferritin) in the IRE lead to a loss of suppression of L-ferritin mRNA translation by IRP, causing up-regulation of ferritin L-chains, independent of iron stores and without iron overload [6,7].

Besides high serum ferritin without iron overload, the only clinical manifestation of HHCS is pediatric cataract formation. Several phenotypes have been described, but the most common is a bilateral cataract with axial and peripheral white flecks with small crystalline aggregates [3,5,8,9]. Cataract formation and symptoms are commonly seen in childhood or adolescence, even though congenital cases have been described previously [5]. Symptoms are usually insidious, progressive, and may even be an incidental finding in a routine ocular examination.

Ferritin reference levels vary but are generally considered to be elevated when larger than 300 ng/mL in males or 200 ng/mL in females [10]. These results should be interpreted with careful consideration of the clinical phenotype, as several medical conditions can lead to elevated ferritin levels. Examples include acute or chronic liver disease, alcohol consumption, systemic inflammatory conditions, injuries, and malignancies [11,12]. Ferritin levels greater than 1000 ng/mL are a non-specific marker of pathology; nonetheless, more than half of the patients with these values are not referred for further investigation when found in routine laboratory tests [13]—evidence for why this matter needs to be further discussed.

Hereditary hemochromatosis (HH) is a genetic disorder, most frequently caused by biallelic mutations in *HFE*, where accumulation of iron in tissues causes direct toxicity by free radicals formation [14]. Unlike HHCS, which does not lead to iron overload, long-term iron deposition in HH can cause cirrhosis, heart failure, adrenal insufficiency, diabetes and polyarthritis [15]. Since HHCS is a rare pathology and less known, patients with family history of hyperferritinemia are frequently misdiagnosed with HH [16]. Therefore, all causes of hyperferritinemia must be kept in mind when evaluating these patients. Supplementary Table S1 lists these possible etiologies and was adapted from Sandnes et al. [12].

Only five Brazilian families have previously been reported with HHCS [17,18,19]. The purpose of this case series is to describe three new Brazilian families with HHCS, as well as to discuss phenotype–genotype correlations. Two of the three novel families here reported also had pathogenic variants in heterozygosis in *HFE.* Possible phenotypic interactions between *FTL* and *HFE* are also considered since both are involved with hyperferritinemia. HHCS is not widely known by ophthalmologists and hematologists; therefore, diagnosis is often delayed, sometimes exposing patients to the invasive and unnecessary procedures usually performed in HH.

## 2. Results

### 2.1. Family 1

#### 2.1.1. Ophthalmological Exam

**Figure 1:**
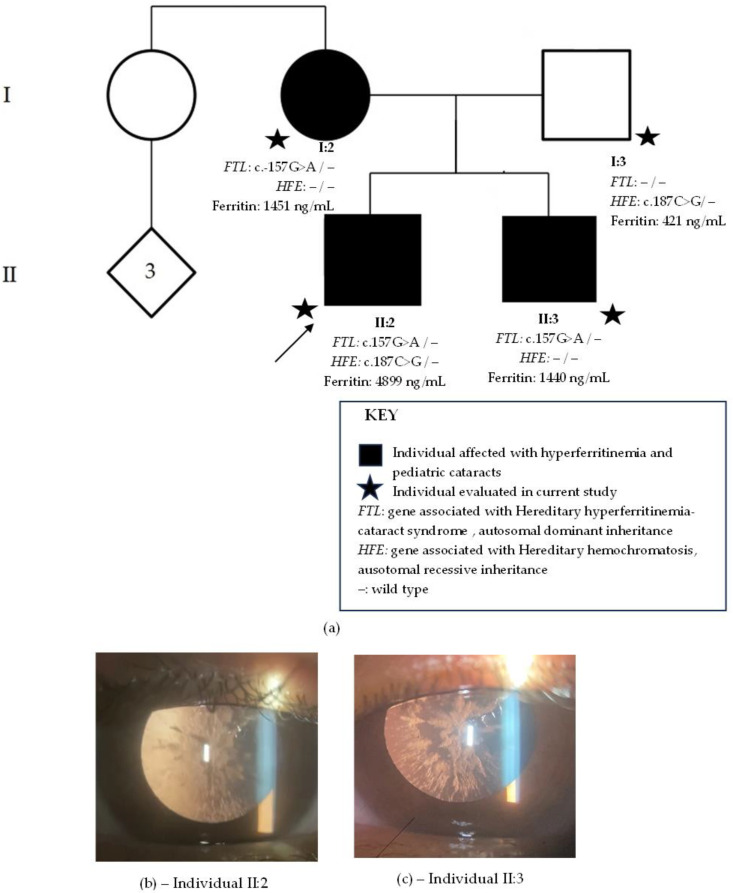
Family 1. (**a**) Family pedigree with two generations depicted (I–II). Circles indicate women, squares men and diamonds unknown gender. Black circles and squares indicate individuals with pediatric cataract. Stars indicate family members evaluated in current study. The arrow indicates the proband. *FTL* is the gene associated with hereditary hyperferritinemia cataracts syndrome with an autosomal dominant inheritance. *HFE* is the gene associated with hereditary hemochromatosis with an autosomal recessive inheritance. (**b**) Sutural cataracts with axial and peripheral white crystalline deposits present in individual II:2. (**c**) Cataract with axial and peripheral white crystalline deposits present in individual II.:3.

The proband (Figure 1a, individual II:2) was examined at age 18. Visual acuity difficulties had been noted by parents at age 4. Slit-lamp biomicroscopy revealed clear corneas and bilateral sutural cataracts with axial and peripheral white crystalline deposits (Figure 1b). Fundoscopy revealed no alterations. His elder brother (Figure 1a, individual II:3) had been discovered to have bilateral cataracts at a routine ophthalmological exam at 9 years old. Visual symptoms only began later, at 14 years old. He was examined at age 25, and slit-lamp biomicroscopy revealed similar bilateral cataracts to his brother, with white axial and peripheral deposits but no sutural component (Figure 1c). Individual II:3 also presented with a normal fundoscopy. Their mother (Figure 1a, individual I-2) received a diagnosis of bilateral cataracts at age 6 and was operated on at age 26. Since she was already pseudophakic at examination and her previous records were unavailable, her cataract phenotype is unknown. The father (Figure 1a, individual I:3) had an unremarkable ophthalmological exam, with no cataracts.

#### 2.1.2. Systemic Family History

Hematological family history revealed that the proband had discovered hyperferritinemia (4899 ng/mL) at a routine examination. Serum iron was of 145 µg/dL, within normal values (75–175 µg/dL). His abdominal MRI with T2-weighted sequences showed no signs of iron overload in the liver. His ferritin levels lowered to 1200 ng/mL after a change in diet, multiple phlebotomies, and a prescription of oral iron chelator. The elder brother also had hyperferritinemia (1440 ng/mL) as well as their father (421 ng/mL) and mother (1451 ng/mL). The family resided in a city in the metropolitan region of Rio de Janeiro city and was unaware of their ancestry.

#### 2.1.3. Whole-Exome Sequencing

Whole-exome sequencing revealed in all family members affected with cataract the heterozygous-likely pathogenic variant, c.-157G>A, in *FTL*. The unaffected father had no relevant genomic alterations in *FTL*. The proband and the father presented with the heterozygous-likely pathogenic variant, c.187C>G (p.H63D), in *HFE*, but not the mother and elder son.

### 2.2. Family 2

#### 2.2.1. Ophthalmological Exam

**Figure 2:**
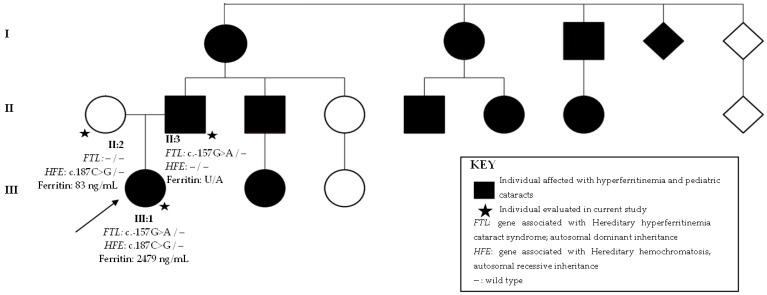
Family 2 pedigree with three generations depicted (I–III). Circles indicate women, squares men and diamonds unknown gender. Black circles and squares indicate individuals with both pediatric cataract and hyperferritinemia. Stars indicate family members evaluated in current study. The arrow indicates the proband. *FTL* is the gene associated with hereditary hyperferritinemia cataracts syndrome with an autosomal dominant inheritance. *HFE* is the gene associated with hereditary hemochromatosis with an autosomal recessive inheritance. U/A: unavailable.

At age three, parents of the proband (Figure 2, individual III:1) noted a head position. Previous ophthalmological exams were normal. Slit-lamp examination revealed bilateral axial posterior subcapsular cataracts. Visual acuity was 0.5 in the right and left eye. She had a physiologic hypermetropia for her age and normal fundoscopy. Her father (Figure 2, individual II:3) had bilateral cataracts diagnosed at age 18, but he referred symptoms beginning at 12 years old. Phacoemulsification was performed at 23 years old. Since he was already pseudophakic at examination, and his previous records were unavailable, his cataract phenotype is unknown. The mother (Figure 2, individual II:2) had an unremarkable ophthalmological exam, with no cataracts.

#### 2.2.2. Systemic Family History

The first-degree cousin had cataracts and hyperferritinemia, which prompted investigation of the proband and father. The proband presented hyperferritinemia (2479 ng/mL), but the father was unavailable for systemic ferritin testing. Both denied previous treatments of hyperferritinemia. The proband also presented with Hirschprung’s disease at birth. The mother had a serum ferritin value of 83 ng/mL, within normal values. Family history revealed three generations with multiple individuals affected by both hyperferritinemia and pediatric cataracts (Figure 2). Other family members with a history of pediatric cataract and hyperferritinemia were unavailable for examination, and it was inferred that they had HHCS. The family resided in Rio de Janeiro city and their history revealed a Native American and Dutch ancestry.

#### 2.2.3. Whole-Exome Sequencing

Whole-exome sequencing revealed in the proband and affected father the heterozygous-likely pathogenic variant, c.-157G>A, in *FTL*. The unaffected mother had no relevant genomic alterations in *FTL*. The heterozygous pathogenic variant c.187C>G (p.H63D) in *HFE* was found in the proband and mother, not in the father.

### 2.3. Family 3

#### 2.3.1. Ophthalmological Exam

**Figure 3:**
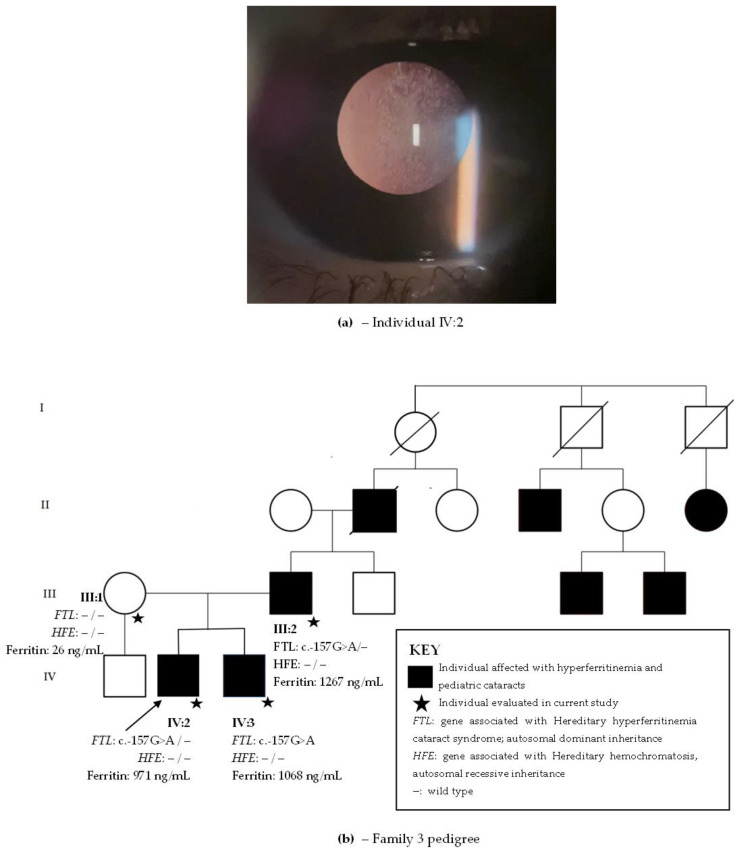
(**a**) Posterior subcapsular cataract in individual IV:2 from family 3. (**b**) Family 3 pedigree with four generations depicted (I–IV). Circles indicate women and squares men. Black circles and squares indicate individuals with both hyperferritinemia and cataract. The arrow indicates the proband. *FTL* is the gene associated with hereditary hyperferritinemia cataracts syndrome with an autosomal dominant inheritance. *HFE* is the gene associated with hereditary hemochromatosis with an autosomal recessive inheritance.

School had noted at age 6 that the proband (Figure 3b, individual IV:2) brought objects closer and he was subsequently diagnosed with bilateral pulverulent cataracts (Figure 3a). His visual acuity was 20/160 in right eye and 20/80 in left eye. No other alterations were found in his ophthalmological exam. Phacoemulsification was performed bilaterally at age 6. His younger brother (Figure 3b, individual IV:3), aged 4, denied visual acuity difficulties and was diagnosed with bilateral posterior subcapsular cataracts at age 4. Visual acuity was 20/70 in the right eye and 20/150 in left eye. The father (Figure 3b, individual II:2) referred low visual acuity symptoms beginning at age 6 and was submitted to phacoemulsification at ages 18 and 30. Since he was already pseudophakic at examination, and his previous records were unavailable, his cataract phenotype is unknown. The mother (Figure 3b, individual III:1) had an unremarkable ophthalmological exam, with no cataracts.

#### 2.3.2. Systemic Family History

The proband, brother, and father had hyperferritinemia (971 ng/mL, 1068 ng/mL, and 1267 ng/mL, respectively). They denied other comorbidities. Three generations of family members presented with hyperferritinemia and pediatric cataract (Figure 3). Clinical information on the eldest generation in the family pedigree is unavailable, but they most likely also presented with hyperferritinemia and cataracts. There were other family members with a history of pediatric cataract and hyperferritinemia which were unavailable for examination, and it was inferred that they probably had HHCS. The family resided in the countryside Rio de Janeiro state and was unaware of their ancestry.

#### 2.3.3. Whole-Exome Sequencing

Whole-exome sequencing revealed the likely pathogenic variant c.-157G>A in *FTL* present in all affected family members with hyperferritinemia and cataracts. Variants in genes causing HH (*HFE*, *BMP2*, *HAMP*, *HJV*, *TFR2*, *SLC40A1*, and *FTH1*) were not found.

Table 1 summarizes the clinical and molecular findings of the eight affected individuals and the three unaffected family members.

## 3. Discussion

The variant c.-157G>A found in all three families was previously reported once in a family of German–Ukrainian ancestry [20]. The proband had bilateral cataracts operated on at age 5 and a serum ferritin level of 2472 ng/mL, compatible with other reports of HHCS. Unfortunately, there is no information on the cataract phenotype to compare with their patients. Only one of the described families in the present article had information on their ancestry, which was of Native American and Dutch origin, unlike the previously described case. The three families were from the state of Rio de Janeiro, one living in the capital, a large city with 6.7 million inhabitants, the other in the metropolitan region, and the third in the countryside of the state. It is unlikely that they were somehow related.

Cataract phenotype in all patients here reported have been previously related to mutations in *FTL*—that is, slow progressing, bilateral diffuse opacities (such as pulverulent, crystalline, and posterior subcapsular) appearing in the first or second decade of life. None of our eight affected patients had congenital cataracts or cataract phenotypes related to an early great visual damage such as total cataracts, which would be uncharacteristic of HHCS.

**Figure 4:**
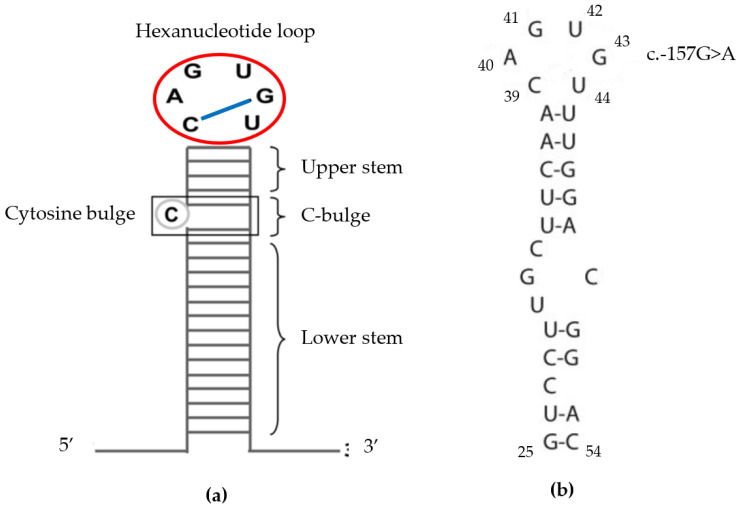
(**a**) The iron responsive element (IRE) is divided into regions—that is, the hexanucleotide loop (marked by the red circle), the upper stem, the Cytosine bulge, and the lower stem. The blue line marks the critical base pair formation, essential for the standard structure of the loop. (**b**) Bases present in the IRE, showing that the loop is composed of nucleotides in positions 39–44, the most highly conserved part of the IRE. The mutation in this report is located at nucleotide position 43. It impairs the critical base pair formation between 39C and 43G. (Adapted from Phillips et al., 2005 [20] and Luscieti et al., 2013 [23].)

About 40 mutations [4], available in Supplementary Table S2, have been described in *FTL.* Attempts have been made to associate disease severity with location of the mutation in the IRE structures [21,22,23]. Higher levels of ferritin were found in patients with mutations located in the hexanucleotide loop or C bulge, as compared to patients with mutations in the upper or lower stems of the IRE (Figure 4) [23]. The variant herein reported is a point mutation which causes a change from guanine to adenosine at position 43 in the loop portion of IRE. The six nucleotides present in this loop (39–44 of the 5′untranslated region) are at the most highly conserved part of IREs, also known as the hexanucleotide loop. This mutation impairs the critical base pair formation between 39C and 43G (dotted line in Figure 4), which is essential for the standard structure of the loop [24,25]. This correlates to our cases since most patients with c.-157G>A (6/8) presented with cataract symptoms before the age of 10 and all of them before 14 years of age.

Causes of cataract development in HHCS patients are still unknown. One theory suggests that oxidative damage caused by disruption in iron homeostasis by ferritin light chain overexpression could cause cataract [26]. Another theory suggests ferritin light chain overexpression would have a strong affinity for the lens due to its high-protein concentration [27]. Supporting this theory, Mumford et al. [28] have shown a larger concentration of L-ferritin in lenses of patients with HHCS, compared to non-HHCS control lenses, as well as staining of the crystalline inclusions with anti-L-ferritin.

Not only did we search for variants in *FTL* in our patients, but also in *HFE*, the gene related to HH, which also causes hyperferritinemia. Unlike HHCS, which has an autosomal dominant mode of inheritance, most forms of HH are inherited in an autosomal recessive pattern. The variant c.187C>G (p.H63D) in *HFE* was present in heterozygosis in four individuals in our cohort; of these, two were affected by HHCS (probands from families 1 and 2). This variant is the second most common in *HFE* and related to less aggressive cases of HH. Accordingly, biallelic mutations in c.187C>G (p.H63D) do not usually develop clinically significant iron overload, unless associated with other iron increasing factors [15]. The most common variant in *HFE* is c.845G>A (p.C282Y), which is related to a severe phenotype when in homozygosis, followed by a more lenient phenotype when in compound heterozygosis with c.187C>G (p.H63D).

Individuals with variants in both *FTL* and *HFE* (probands from families 1 and 2) had the highest levels of ferritin in our cohort (4899 ng/mL and 2479 ng/mL), even though the variant p.H63D in *HFE* is known to cause a more subtle phenotype. The other six individuals with HHCS and no variants in *HFE* had lower levels of ferritin, varying from 971 to 1471 ng/mL. Two individuals without HHCS had variants in *HFE*, and their ferritin levels were mildly elevated (421 ng/mL) and within normal values (83 ng/mL). Considering the above, the question was raised as to if patients with HHCS and variants in heterozygosis in *HFE* would have higher levels of serum ferritin.

Eris et al. [29] strengthen the possibility of genotype–phenotype correlations between *FTL* and *HFE*. They describe a patient with HHCS and the same variant, c.187C>G, (p.H63D) in *HFE*, only in homozygosis. She did not present with iron overload symptoms, which could be related to the fact that her biallelic variants in *HFE* were the least severe of all. Her cataract phenotype was similar to those presented by patients in family 1. Her sons were heterozygous for c.187C>G (p.H63D) and had hyperferritinemia (1209–1962 ng/mL) but did not present cataracts. The proband’s ferritin levels were the highest among her family members (2678 ng/mL). Unfortunately, genetic evaluation of *FTL* in the proband and her three sons were not performed, but we can infer that the mother was probably the only individual with mutations in *FTL*, since she was the only one with pediatric cataracts. Nonetheless, this case series reinforces possible genotype–phenotype correlations when both genes display concurrent mutations since the proband, who probably has mutations in both genes, presented with the highest value of ferritin. Likewise, Eris et al. show that heterozygotes for c.187C>G (p.H63D) can present hyperferritinemia, with values even higher than our heterozygous patients.

Other previous cases of coinheritance of alleles in *FTL* and *HFE* have been described [9,18,19], but without comparing the relationship between HHCS phenotype in individuals with the same variants in *FTL* and *HFE*. It is important to note that heterozygosis in *HFE* is not uncommon in the Caucasian population, and about 25% of individuals are heterozygotic for c.187C>G (p.H63D). Therefore, the prevalence of HFE mutations found in our HHCS patients (36%) is not so distinct from the literature findings. A larger number of patients with coinheritance of alleles in *FTL* and *HFE* would be needed to establish a definitive correlation with ferritin values. Considering the high prevalence of heterozygosis in *HFE* (mainly p.C282Y and p.H63D), it would be interesting to search for variants in this gene in all HHCS patients. It is possible that the more aggressive *HFE* variant (p.C282Y) causes even higher levels of ferritin in HHCS patients.

HHCS is a rare condition, and it is important to raise awareness of it, not only to improve diagnostic yield but to prevent unnecessary and potentially damaging treatments, such as phlebotomies and iron oral chelators, as are frequently described. Therefore, patients with hyperferritinemia and no conclusive diagnosis should be sent to ophthalmological evaluation and variants in *FTL* should be sought. This case series also brings to discussion the possibility that mutations in the *HFE* gene increase ferritin levels of patients with HHCS. Therefore, we suggest that *HFE* variants be screened in patients with HHCS in order to further evaluate this hypothesis.

## 4. Materials and Methods

### 4.1. Study Design

A case series with clinical description and next-generation sequencing of 11 individuals from 3 distinct families with a history of autosomal dominant pediatric cataract and hyperferritinemia. Of the 11 subjects, 3 were asymptomatic (1 from each family), and trio analysis was performed.

### 4.2. Clinical Evaluation

The ophthalmic exam was performed by an ophthalmologist via slit-lamp biomicroscopy, intraocular pressure (IOP), and indirect ophthalmoscopy in all individuals. Individuals not yet submitted to cataract surgery had their cataract phenotypes documented. Genetic evaluation of family history and pedigree, as well as detailed review of previous laboratorial and hematological clinical records, were performed by a clinical geneticist. History of congenital TORCH infections (toxoplasmosis, rubella, cytomegalovirus, herpes simplex, syphilis, varicella zoster, zika), use of corticosteroids and ocular trauma were ruled out.

### 4.3. Genomic DNA

Eleven family members had their peripheral blood samples collected in EDTA tubes. PureLink^®^ Genomic DNA Mini Kit Thermofisher (Waltham, MA, USA) was used to extract genomic DNA from peripheral blood leukocytes according to the manufacturer’s protocol. Invitrogen Qubit^®^ 4 Fluorometer Thermofisher (Waltham, MA, USA) was used to determine DNA concentration in samples. To assess DNA purity, a Spectrophotometer NanoDrop^®^ 2000 was used to evaluate the ratio of the absorbance at 260/280 nm (average of 1.90 for all samples) and at 260/230 nm (average of 1.91 for all samples). DNA samples were stored at 4 °C prior to use.

### 4.4. Library Preparation and Whole-Exome Sequencing

Illumina DNA Prep with Enrichment (16 samples) was used to prepare DNA libraries according to the manufacturer’s instructions (Illumina, Inc., San Diego, CA, USA). Illumina Exome Panel was used, which covers 37.5 Mb coding content (≥99% of RefSeq, CCDS, ClinVar and ACMG pathogenic/likely pathogenic variants, COSMIC Cancer Gene Census). Sequencing was performed using NVSEQ 6000 S4 Rgt Kit v1.5 (200cyc). During library preparation, DNA fragments of 400 bp long on average were evaluated using the Bioanalyzer by Agilent.

### 4.5. Bioinformatics Analysis

Sequencing data were processed using the nf-core pipeline Sarek aligned to the human reference genome GRCh37 [30]. The generated VCF files were analyzed using Franklin^®^ by Genoox platform (Israel), and the genetic variant calls were performed against the reference sequence of hg38 from the University of California Santa Cruz (UCSC) (Santa Cruz, CA, USA) Genome Browser. The analysis strategy started with a virtual panel based on Human Phenotype Ontology (HPO), where 658 genes were found related to cataract in general. Genogram trio analysis was performed on affected and unaffected family members using the trio analysis tools available on Franklin^®^. During variant interpretation, we considered allele frequency using the Exome Aggregation Consortium database (ExAC), 1000 Genomes Project database, gnomAD and ABraOM, an online archive of Brazilian mutations [31]. Twelve predictors were considered for pathogenicity: BayesDel_addAF, DANN, DEOGEN2, EIGEN, FATHMM-MKL, LIST-S2, M-CAP, MVP, MutationAssessor, MutationTaster, SIFT, and PrimateAI. The clinical significance of variants was evaluated with ClinVar, Polymorphism database (dbSNP), and Human Gene Mutation Database (HGMD). Cat-Map, an online chromosome map and reference database for cataract in humans and mice [4], was also searched for previous variant descriptions and clinical associations. Variant naming was based on the reference sequence NM_000146.

## Figures and Tables

**Table 1:** Clinical and molecular findings in eight individuals with hereditary hyperferritinemia-cataract syndrome (HHCS) and thee family members without HHCS.

Family	Individual	Cataract (Yes:Y/ No: N)	Age of Symptoms (Years)	Age of Surgery (Years)	Bilateral Cataract Phenotype	Ferritin (ng/mL)	*FTL* Mutation in Heterozygosis	*HFE* Mutation in Heterozygosis
1	II:2	Y	4	Phakic	Sutural with axial and peripheral white crystalline deposits	4899	c.-157G>A	c.187C>G
1	II:3	Y	14	Phakic	Axial and peripheral white crystalline deposits	1440	c.-157G>A	Absent
1	I:2	Y	6	26	Unknown	1451	c.-157G>A	Absent
1	I:3	N	N/A	N/A	N/A	421	Absent	c.187C>G
2	III:1	Y	3	Phakic	Posterior subcapsular	2479	c.-157G>A	c.187C>G
2	II:3	Y	12	23	Unknown	U/A	c.-157G>A	Absent
2	II:2	N	N/A	N/A	N/A	83.9	Absent	c.187C>G
3	IV:2	Y	6	6	Pulverulent	971	c.-157G>A	Absent
3	IV:3	Y	4	Phakic	Posterior subcapsular	1068	c.-157G>A	Absent
3	III:2	Y	6	18 and 30	Unknown	1267	c.-157G>A	Absent
3	III:1	N	N/A	N/A	N/A	26	Absent	Absent

Y: yes; N: No; N/A: non applicable; U/A: unavailable; Phakic: patient still has his/her natural lens and was not yet operated on.

## Data Availability

Data are available on the public online database Cat-Map and from related publications and supplements. This data can be found here: https://cat-map.wustl.edu/, accessed on 22 May 2023.

## Supplementary Materials

The supporting information can be downloaded at: https://www.mdpi.com/article/10.3390/ijms241511876/s1.

## References

[1] Bonneau D., Winter-Fuseau I., Loiseau M.-N., Amati P., Berthier M., Oriot D., Beaumont C. (1995). Bilateral cataract and high serum ferritin: A new dominant genetic disorder?. J. Med. Genet..

[2] Girelli D., Olivieri O., De Fransceschi L., Corrocher R., Bergamaschi G., Cazzola M. (1995). A linkage between hereditary hyperferritinaemia not related to iron overload and autosomal dominant congenital cataract. Br. J. Haematol..

[3] Chang-Godinich A., Ades S., Schenkein D., Brooks D., Stambolian D., Raizman M. (2001). Lens Changes in Hereditary Hyperferritinemia–Cataract Syndrome. Am. J. Ophthalmol..

[4] Cat-Map. https://cat-map.wustl.edu/.

[5] Craig J.E., Clark J.B., McLeod J.L., Kirkland M.A., Grant G., Elder J.E., Toohey M.G., Kowal L., Savoia H.F., Chen C. (2003). Hereditary Hyperferritinemia-Cataract Syndrome—Prevalence, lens morphology, spectrum of mutations and clinical presentations. Arch. Ophthalmol..

[6] Aslan D., Akata R.F., Atalay H.T., Üçgül A.Y. (2019). Elevated serum ferritin level with cataract of spectacular morphology: Hyperferritinemia cataract syndrome. Pediatr. Hematol. Oncol..

[7] Roetto A., Bosio S., Gramaglia E., Barilaro M.R., Zecchina G., Camaschella C. (2002). Pathogenesis of hyperferritinemia cataract syndrome. Blood Cells Mol. Dis..

[8] Christiansen G., Mohney B.G. (2007). Hereditary hyperferritinemia-cataract syndrome. J. AAPOS.

[9] Moravikova J., Honzik T., Jadvidzakova E., Zdrahalova K., Pourova R.K., Korbascova M., Liskova P., Dudakova L. (2020). Hereditary hyperferritinemia-cataract syndrome in three Czech families: Molecular genetic testing and clinical implications. J. AAPOS.

[10] Elsayed M.E., Sharif M.U., Stack A.G. (2016). Transferrin saturation: A body iron biomarker. Advances in Clinical Chemistry.

[11] Adams P. (2008). Management of elevated serum ferritin leves. Gastroenterol. Hepatol..

[12] Sandnes M., Ulvik R.J., Vorland M., Reikvam H. (2021). Hyperferritinemia—A clinical overview. J. Clin. Med..

[13] Ogilvie K., Fitzsimons K., Fitzsimons E.J. (2010). Serum ferritin values in primary care: Are high values overlooked?. J. Clin. Pathol..

[14] Papanikolaou G., Pantopoulos K. (2005). Iron metabolism and toxicity. Toxicol. Appl. Pharmacol..

[15] Murphree C.R., Nguyen N.N., Raghunathan V., Olson S.R., DeLoughery T., Shatzel J.J. (2020). Diagnosis and management of hereditary haemochromatosis. Vox Sang..

[16] Nos F.C., Hernández G., Ferrer-Cortès X., Hernandez-Rodriguez I., Navarro-Almenzar B., Fuster J.L., Cortés M.B., Pérez-Montero S., Tornador C., Sanchez M. (2021). Hereditary Hyperferritinemia Cataract Syndrome: Ferritin L Gene and Physiopathology behind the Disease- Report of New Cases. Int. J. Mol. Sci..

[17] Alvarenga A.M., da Silva N.K., Cançado R.D., de Carvalho L.E.M.R., Santos P.C.J.L. (2022). Brazilian family with hyperferritinemia-cataract syndrome: Case report. Einstein.

[18] Meneses F.G.A., Schnabel B., Silva I.D.C.G. (2011). Identification of the mutations associated with hereditary hyperferritinemia cataract syndrome and hemocromatosis in a Brazilian family. Clin. Genet..

[19] Petroni R.C., da Rosa S.E.A., de Carvalho F.P., Santana R.A.F., Hyppolito J.E., Nascimento C.M.D.B., Hamerschlak N., Campregher P.V. (2017). Ferritin light chain gene mutations in two Brazilian families with hereditary hyperferritinemia-cataract syndrome. Einstein.

[20] Phillips J.D., Warby C.A., Kushner J.P. (2005). Identification of novel mutation in the L-ferritin IRE leading to hereditary hyperferritinemia-cataract syndrome. Am. J. Med. Genet..

[21] Allerson C.R., Cazzola M., Rouault T.A. (1999). Clinical severity and thermodynamic effects of iron-responsive element mutations in hereditary hyperferritinemia-cataract syndrome. J. Biol. Chem..

[22] Cazzola M., Bergamaschi G., Tonon L., Arbustini E., Grasso M., Vercesi E., Barosi G., Bianchi P.E., Cairo G., Arosio P. (1997). Hereditary hyperferritinemia-cataract syndrome.: Relationship between phenotypes and specific mutations in the iron-responsive element of ferritin light-chain mRNA. Blood.

[23] Luscieti S., Tolle G., Aranda J., Campos C.B., Risse F., Morán É., Muckenthaler M.U., Sánchez M. (2013). Novel Mutation in the ferritin-L iron-responsive element that only mildly impair IRP binding cause hereditary hyperferritinaemia cataract syndrome. Orphanet J. Rare Dis..

[24] Addess K.J., Basilion J.P., Klausner R.D., Rouault T.A., Pardi A. (1997). Structure and Dynamics of the Iron ResponsiveElement RNA: Implications for Binding of the RNA by Iron Regulatory Binding Proteins. J. Mol. Biol..

[25] Ke Y., Wu J., Leibold E.A., Walden W.E., Theil E.C. (1998). Loops and bulge/loops in iron-responsive element isoforms influence iron regulatory protein binding: Fine tuning of mRNA regulation?. J. Biol. Chem..

[26] Levi S., Girelli D., Perrone F., Pasti M., Beaumont C., Corrocher R., Albertini A., Arosio P. (1998). Analysis of ferritins in lymphoblastoid cell lines and in the lens of subjects with hereditary hyperferritinemia-cataract syndrome. Blood.

[27] Shekunov J., de Groen P.C., Lindor N.M., Klee G.G., Aleff R.A., Wieben E.D., Mohney B.G. (2011). Hereditary hyperferritinemia-cataract syndrome in two large multigenerational American families. J. AAPOS.

[28] Mumford A.D., Cree I.A., Arnold J.D., Hagan M.C., Rixon K.C., Harding J.J. (2000). The lens in hereditary hyperferritinaemia cataract syndrome contains crystalline deposits of L-ferritin. Br. J. Ophthalmol..

[29] Eris T., Yanik A.M., Demirtas D., Yilmaz A.F., Toptas T. (2023). Hereditary Hyperferritinemia-Cataract Syndrome in a Family With HFE-H63D Mutation. Cureus.

[30] Garcia M., Juhos S., Larsson M., Olason P.I., Martin M., Eisfeldt J., DiLorenzo S., Sandgren J., De Ståhl T.D., Ewels P. (2020). Sarek: A portable workflow for whole-genome sequencing analysis of germline and somatic variants. F1000Research.

[31] ABraOM: Brazilian Genomic Variants. http://abraom.ib.usp.br/.

