# Supplementary material for: Genotypic–Phenotypic Correlations of Hereditary Hyperferritinemia-Cataract Syndrome: Case Series of Three Brazilian Families
Source: Int J Mol Sci. 2023 Jul 25;24(15):11876. doi: 10.3390/ijms241511876 (PMC10419074; doi:10.3390/ijms241511876)
Supplement: Supplementary file 1 [file ijms-24-11876-s001.zip › Supplementary table S1 - causes of hyperferritinemia.pdf]

Supplementary Table S1: Hyperferritinemia causes in conditions with and without iron overload (adapted from Sandnes, *J Clin Med*, 2021 [12]).

Hyperferritinemia without iron overload	**Common causes**<br>Cellular damage<br>Metabolic syndrome and obesity<br>Insulin resistance / diabetes mellitus<br>Excessive alcohol consumption<br>Inflammatory and infectious conditions<br>Malignancy (solid and hematological)<br>**Rare causes**<br>Benign hyperferritinemia / Hereditary hyperferritinemia cataract-syndrome<br>Immune -mediated syndomes (primary and secondary HLH, adult onset Still's disease)<br>Gaucher disease
Hyperferritinemia with or without iron overload	Chronic liver disease (cirrhosis, alcoholic liver disease, NAFLD, viral hepatitits, porphyria cutanea tarda)
Hyperferritinemia with iron overload	**Common causes**<br>*HFE* hemochromatosis<br>Dysmetabolic iron overloading syndrome<br>Iron-loading anemias (congenital or acquired)<br>Iatrogenic iron overload (RBC transfusion, parenteral iron administration)<br>African iron overload<br>**Rare causes**<br>Non-*HFE* hereditary hemochromatosis<br>Ferroportin disease<br>Aceruplasminemia / hypoceruplasminemia<br>Atransferrinemia / hypotransferrinemia

HLH, hemophagicytic lymphohistiocytosis; NAFLD, non-alcoholic fatty liver disease; RBC, red blood cell.
