# Supplementary material for: Genotypic–Phenotypic Correlations of Hereditary Hyperferritinemia-Cataract Syndrome: Case Series of Three Brazilian Families
Source: Int J Mol Sci. 2023 Jul 25;24(15):11876. doi: 10.3390/ijms241511876 (PMC10419074; doi:10.3390/ijms241511876)
Supplement: Supplementary file 1 [file ijms-24-11876-s001.zip › Supplementary table S2 - mutations in FTL 24-06.pdf]

Supplementary Table S2: Variants in *FTL* previously related to Hereditary Hyperferritinemia-cataract syndrome. (source: Catmap, public online database available on: https://cat-map.wustl.edu/, access 1 May 2023.

Region	DNA change	Origin
Promoter	c.-415C>A	Italy
Exon 1 (IRE)	c.-220_-196del25	Australia
Exon 1 (IRE)	c.-193C>G	Italy
Exon 1 (IRE)	c.-190_-162del29	Italy
Exon 1 (IRE)	c.-186C>G	Unknown
Exon 1 (IRE)	c.-182C>A	Germany
Exon 1 (IRE)	c.-182C>T	Italy
Exon 1 (IRE)	c.-178T>G	Germany, Italy
Exon 1 (IRE)	c.-178_-173del6	Italy
Exon 1 (IRE)	c.-176T>C	Germany, Switzerland
Exon 1 (IRE)	c.-174T>G	Germany
Exon 1 (IRE)	c.-171C>G	Italy, Norway, Spain
Exon 1 (IRE)	c.-168G>A	Italy, India, Germany, USA, Brazil, Spain
Exon 1 (IRE)	c.-168G>C	Australia, Thailand/UK, Romania, Switzerland, Italy, Czech Republic
Exon 1 (IRE)	c.-168G>T	Italy, France, Australia, UK, Israel, USA, Germany, Spain, Korea
Exon 1 (IRE)	c.-168_-165delGCTT	Canada
Exon 1 (IRE)	c.-167C>A	France, Switzerland
Exon 1 (IRE)	c.-167C>T	Spain, USA, UK, Ireland, Italy, Australia, Czech Republic, China
Exon 1 (IRE)	c.-166T>C	France
Exon 1 (IRE)	c.-164C>A	UK
Exon 1 (IRE)	c.-164C>G	Brazil
Exon 1 (IRE)	c.-164C>T	Spain
Exon 1 (IRE)	c.-164_-158delCAACAGT	Spain
Exon 1 (IRE)	c.-163A>C	Italy
Exon 1 (IRE)	c.-163A>G	Unknown
Exon 1 (IRE)	c.-163A>T	Spain
Exon 1 (IRE)	c.-162_-161delAC	Canada
Exon 1 (IRE)	c.-161delC	Spain
Exon 1 (IRE)	c.-161C>A	Italy, Australia
Exon 1 (IRE)	c.-161C>G	France, Greece
Exon 1 (IRE)	c.-161C>T	UK, Italy, Spain, Czechia
Exon 1 (IRE)	c.-160A>G	France, Italy, UK, Spain, Germany, Turkey
Exon 1 (IRE)	c.-159G>C	Italy, Brazil
Exon 1 (IRE)	c.-158_-143del16	France
Exon 1 (IRE)	c.-157G>A	Germany/Ukraine
Exon 1 (IRE)	c.-154T>G	Italy
Exon 1 (IRE)	c.-153G>A	France
Exon 1 (IRE)	c.-153G>C	Finland
Exon 1 (IRE)	c.-152G>T	Finland
Exon 1 (IRE)	c.-151A>C	Italy
Exon 1 (IRE)	c.-151A>G	Belgium
Exon 1 (IRE)	c.-149G>C	Canada, USA
Exon 1 (IRE)	c.-148G>C	Germany
Exon 1 (IRE)	c.-144A>T	France
